# New Organic Salt from Levofloxacin-Citric Acid: What Is the Impact on the Stability and Antibiotic Potency?

**DOI:** 10.3390/molecules27072166

**Published:** 2022-03-27

**Authors:** Ilma Nugrahani, Agnesya Namira Laksana, Hidehiro Uekusa, Hironaga Oyama

**Affiliations:** 1 School of Pharmacy, Bandung Institute of Technology, Bandung 40132, Indonesia; agnesyanamira@gmail.com; 2 Department of Chemistry, School of Science, Tokyo Institute of Technology, Tokyo 152-8551, Japan; uekusa@chem.titech.ac.jp (H.U.); oyama.h.ab@m.titech.ac.jp (H.O.)

**Keywords:** organic salt, levofloxacin, citric acid, levofloxacin-citrate, stability, antibiotic potency

## Abstract

This research dealt with the composition, structure determination, stability, and antibiotic potency of a novel organic salt composed of levofloxacin (LF) and citric acid (CA), named levofloxacin-citrate (LC). After a stoichiometric proportion screening, the antibiotic-antioxidant reaction was conducted by slow and fast evaporation methods. A series of characterizations using thermal analysis, powder X-ray diffractometry, vibrational spectroscopy, and nuclear magnetic resonance confirmed LC formation. The new organic salt showed a distinct thermogram and diffractogram. Next, Fourier transform infrared indicated the change in *N*-methylamine and carboxylic stretching, confirmed by ^1^H nuclear magnetic resonance spectra to elucidate the 2D structure. Finally, single-crystal diffractometry determined LC as a new salt structure three-dimensionally. The attributive improvements were demonstrated on the stability toward the humidity and lighting of LC compared to LF alone. Moreover, the antibiotic potency of LF against *Staphylococcus aureus* (Gram-positive) and *Escherichia coli* (Gram-negative) enhanced ~1.5–2-fold by LC. Hereafter, LC is a potential salt antibiotic-antioxidant combination for dosage formulas development.

## 1. Introduction

For decades, the multicomponent solid structure has been developed widely as the primary strategy to modulate the performance of active pharmaceutical ingredients (API) [1]. The combination compound may involve ionic and nonionic bonding [2,3], which, in some cases, are tailored for a particular purpose, such as increased solubility and stability [3,4], also improving the unpleasant taste [5]. 

Generally, antibiotics have poor stability [6], i.e., ciprofloxacin, a fluoroquinolone antibiotic, can be improved by chemical derivatization or solid-state development. Recently, we composed and reported ciprofloxacin salicylate, 1.75 hydrates, that modulated the solubility [7]. However, the development of fluoroquinolone antibiotics has generated some derivates, including levofloxacin (LF), which exhibits a broader spectrum of antibacterial activity [8] and better physicochemical properties. LF is assigned to Class I in the current Biopharmaceutics Classification System (BCS), which has good solubility and oral bioavailability [9]. However, LF was reported to degrade under lighting [10]. Therefore, some efforts have been made to enhance this antibiotic’s stability by combining this antibiotic with other compounds. For example, the drug–drug multicomponent of LF, metacetamol [4], and drug–excipient multicomponent of LF, stearic acid [5], have been reported to improve the physicochemical properties, in addition to masking the taste and aroma [4,5]. 

**Figure 1:**
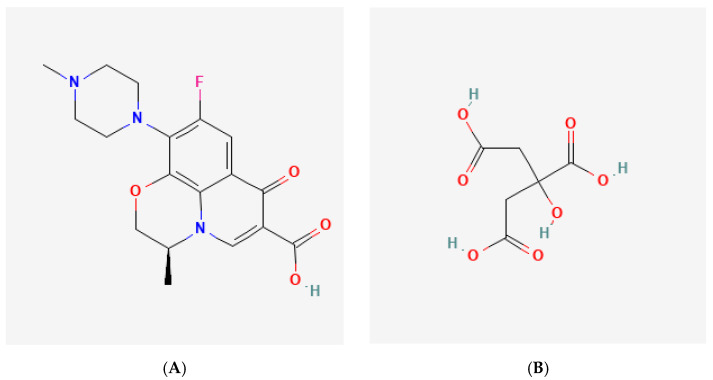
Molecular structure of parent compounds: (**A**) levofloxacin (LF) [13] and (**B**) citric acid (CA) [14].

This research aimed to derive a new composition from LF with an antioxidant, citric acid (CA), named levofloxacin-citrate (LC), and then to comprehensively investigate its structure, stability, and antibiotic potency. CA was chosen as the reactant due to the following reasons. First, it has four hydrogen bonding sites [11] more than its counterparts, i.e., acetic acid and oxalic acid. Secondly, CA is a stable carboxylic derivate under ambient conditions. Finally, this acid is safe and can act as an antioxidant with a minor antibiotic potency [12]. Hence, CA was expected to interact with LF easily, modulate the chemical stability of LF, and improve the potency at the same time. The molecular structures of LF and CA are shown in Figure 1, revealing some potential binding sites, such as carboxylic acid, ketone, amine, and flour in the LF structure; and a hydroxy besides the three carboxylate moieties in CA as the counterpart. 

The experiment started with the molar ratio determination by a phase diagram composition, followed by LC preparation under two methods, fast evaporation (FE) and slow evaporation (SE). All products were then characterized by thermal analysis using electrothermal analysis and differential scanning calorimetry (DSC) and powder X-ray diffractometry (PXRD). Next, the structural study was performed by Fourier transform infrared (FTIR) and ^1^H nuclear magnetic resonance (^1^H NMR). Afterward, an appropriate single crystal was analyzed using single-crystal X-ray diffractometry (SCXRD) to determine the structure entirely. Stability and antibiotic potency tests of LC were then conducted to provide comprehensive data. The potency test measured the minimum inhibitory concentration (MIC) compared to LF alone and the physical mixture (PM). 

## 2. Materials and Methods

### 2.1. Materials 

This experiment used LF-(S)-enantiomer pro-analysis in hemihydrate form (Sigma Aldrich, Jakarta, Indonesia), which is hereafter referred to as levofloxacin (LF); pharmaceutical-grade LF from PT. Kimia Farma (Bandung, Indonesia); CA pro-analysis from Chemical Planets (Depok, Indonesia); potassium bromide/KBr for infrared analysis (Merck, Jakarta, Indonesia); and 95% ethanol, methanol 95%, and distilled water from Sakura Medical (Bandung, Indonesia). Buffer pH 6.8, buffer pH 7.4, and buffer pH 1.2 were prepared at Bandung Institute of Technology (Bandung, Indonesia) for the antibiotic potency study; KH_2_PO_4_, Na_2_HPO_4_, natrium chloride, natrium hydroxide, and HCl 37% were from PT. Bratachem (Bandung, Indonesia); aluminum plates and lids were used for thermal analysis (Rigaku, Tokyo, Japan); a capillary tube was used for electrothermal analysis from CV. Prima Medicha (Bandung, Indonesia); CDCl_3_ and D_2_O were used as solvents for ^1^H NMR characterization, purchased from Sigma Aldrich (Jakarta, Indonesia); and Karl Fischer Aquastar Combi-Titrant 5 reagent was purchased from E. Merck (Darmstadt, Germany). The bacteria used in this experiment were Staphylococcus aureus ATCC 25953 and Escherichia coli ATCC 9001, prepared by Microbiology Laboratory, School of Pharmacy, Bandung Institute of Technology, Indonesia. Meanwhile, the bacteria growth medium and water used for the potency test were nutrient agar (Merck, Jakarta, Indonesia), brain heart infusion broth (Oxoid, Jakarta, Indonesia), Mueller Hinton broth (Oxoid, Jakarta, Indonesia), Mueller Hinton agar (Oxoid, Jakarta, Indonesia), plate count agar (Merck, Jakarta, Indonesia), physiological sodium chloride solution (Merck, Jakarta, Indonesia), 0.5 McFarland standard solution (bioMerieux, Jakarta, Indonesia), and sterile distilled water (School of Pharmacy, Bandung Institute of Technology, Indonesia).

### 2.2. Methods 

Screening the Optimal Molar Ratio of a Multicomponent System Using a Binary Phase Diagram.

Molar ratios of LF-CA: 10:0, 8:2, 7:3, 6:4, 5:5, 4:6, 3:7, 2:8, and 0:10 were weighed using a digital scale (Fujitsu FSR-A220, Tokyo, Japan) and were mixed thoroughly. After the mixture was homogeneous, electrothermal analyzer (Electrothermal AZ 9003, Staffordshire, UK) and DSC Rigaku Thermoplus EVO2-DSC8231 (Tokyo, Japan) measured the melting point and thermal profile, respectively. A binary phase diagram was then composed by plotting the melting point (*y*-axis) against the mole fraction of LF (*x*-axis).

#### 2.2.1. LC Making

The multicomponent system was made using fast evaporation (FE) and slow evaporation (SE). 

LF (1 meq) was dissolved into a minimum amount of methanol or ethanol 70–80% in water, ~25 mL, at room temperature. Next, CA (1 meq) was added to the solution, mixed until transparent, and filtered. In the fast evaporation method, the solution was dried up at 70–80 °C; meanwhile, in the slow evaporation, the solution was recrystallized slowly from water solution at room temperature to produce a single crystal. First, the white crystal/powder was characterized using solid analysis instruments: electrothermal analyzer, DSC, and PXRD; then, its 2D structure was analyzed using FTIR and ^1^H NMR. Next, an appropriate single crystal from the slow evaporation method was 3D structurally determined using SCXRD. Finally, the physical mixture (PM) was prepared from a homogenous mixture of LF and CA, where each size of the compound was controlled to the same size using an 18-mesh sieve.

#### 2.2.2. Solid Characterization and Structure Determination 

Each starting material and LC system were observed visually using a binocular microscope (Olympus CX21, Tokyo, Japan). An appropriate single crystal was used for structure determination using SCXRD. The melting point and thermal profile were then measured with electrothermal analyzer (Electrothermal AZ 9003, Staffordshire, UK) and DSC (Rigaku Thermoplus EVO2 DSC8231, Tokyo, Japan). PXRD Rigaku Miniflex (Tokyo, Japan) was used to analyze the crystal phase. Meanwhile, FTIR Jasco 4200 Type-A (Oklahoma City, OK, USA) was used to read the structure change. Afterward, ^1^H NMR NMReady-60 by Nanalysis (Calgary, Canada) observed the 2D structure. Finally, the final structural study was performed using an SCXRD XtaLAB Synergy-DW, Rigaku OD (Tokyo, Japan).

#### 2.2.3. Electrothermal Melt Range Measurement

A small sample was inserted into the capillary tube until a height of about 1–2 mm, tapped entirely, then placed in the electrothermal sample holder. The starting temperature was adjusted at 30 °C with a heating rate of 10 °C/min, then observed through the viewer hole. The transition temperature was recorded until the sample wholly melted.

#### 2.2.4. Observation of Multicomponent System Forms 

The shapes of LF, CA, and LC crystals were observed under a binocular microscope without a cover glass at 100× magnification. 

#### 2.2.5. DSC Analysis

Sample powder as much as 1–3 mg was put into an aluminum pan, then closed with a lid and packed using a crimping tool. An empty aluminum pan was used as the reference. After that, the sample and reference pans were inserted into the DSC, which had been calibrated using indium as the standard of measurement. Analysis was carried out in a temperature range from 30 to 250 °C for LC and LF and 30 to 170 °C for CA, with the heating rate set at 10 °C/min. Then, the data were processed in Microsoft Excel 365 software.

#### 2.2.6. FTIR Analysis

Sample powder and KBr were mixed in a ratio of 1:100 and crushed in a marble mortar until homogeneous. The mixture was then compressed using a hydraulic press to form a transparent plate and inserted into the FTIR sample holder. The measurement was conducted at a wavenumber resolution of 4000–400 cm^−1^. The LC spectrum was compared to LF, CA, and their physical mixtures (PM), based on the data processed with Microsoft Excel 365 software.

#### 2.2.7. NMR Analysis 

The 2D structure of LC was determined using ^1^H NMR. The ^1^H NMR spectrum of samples was observed to characterize the functional groups available. The LF and CA sample was dissolved with CDCl_3_; meanwhile, the LC sample was dissolved with D_2_O. Each sample solution contained tetramethylsilane (TMS) as an internal reference per the following conditions: spectral width 735.29 Hz (−0.2–13 ppm), digital resolution 0.179 Hz/pt, and relaxation delay 1.929335 s. The spectrum calculation was collected and processed using (Calgary, AB, Canada) Nanalysis NMReady v2.0.7 software. 

#### 2.2.8. PXRD Analysis

The powder sample was placed in a sample holder between the Mylar films. Tests were carried out with PXRD in the measurement at 2θ with intervals of 3–40° and a scan speed of 0.01–3 °/min, using Cu-Kα radiation with a graphite monochromator. The device was operated using a voltage of 40 kV and a current of 35 mA. The PXRD pattern was plotted using Microsoft Excel 365 software.

#### 2.2.9. SCXRD Analysis

A microscope was used to select a suitable single crystal from the SE method and was then put in a sample holder for SCXRD. The data were collected in ω-scan mode using a Cu Kα radiation (λ = 1.54184 Å) rotating anode source under −180 °C. The collected data were integrated and scaled using (Rigaku, Tokyo, Japan) CrysAlis^Pro^ software. The crystal structure was solved directly by SHELXT and refined with SHELXL. The hydrogen atoms were found in a different Fourier map, placed by geometrical calculations using a riding model during the refinement. Meanwhile, the nonhydrogen atoms were refined anisotropically. Finally, the structure graph was composed using the Mercury 4.3.1 program.

#### 2.2.10. Stability Test

The simple stability test was performed by storing the samples in open air under direct sunlight for one month. Then, the hygroscopicity and photo-degradation were observed.

- Hygroscopicity

For a simple hygroscopicity study, 1 g of levofloxacin hemihydrate (LFH) and LC were put in a watch glass under open air of ambient conditions (70–80% RH/25 ± 2 °C). The fresh samples were placed directly on a watch glass under open air of ambient conditions (70–80% RH/25 ± 2 °C). Then, the sampling was performed twice a week for four weeks of observation. The water content was measured by the Karl Fischer titration apparatus Mettler Toledo V20 (Giessen, Germany) using Aquastar Combi-Titrant 5 reagent. Previously, one milliliter of reagent was determined to equal 4.7 mg of water. Twenty-five milligrams of each sample (LF and LC) was carefully weighed and then added to the titrator chamber that contained reagents. The water content measurement started after the drift value was less than 25 µg/min and finished once the stable amount of water was displayed.

- Photo-degradation

For photolytic degradation, the samples equal to 1 g of LF were exposed to sunlight for four weeks in a closed transparent vial and were then used for the study. During the sample’s exposure to sunlight, the humidity and temperature were recorded under the range of ambient conditions in Bandung, Indonesia (70–80% RH/25 ± 2 °C). Sampling was performed every week, and the chemical stability was investigated by determining the LF level in the samples using spectrophotometry UV-Vis by the Beckman DU640 UV/Vis Spectrophotometer (Indianapolis, Indiana, USA) at wavelength λ = 288 nm after reducing the sample’s weight with the water content, which was determined by Karl Fischer titration. Samples were dissolved in distilled water. The content of LF in the sample was determined using a verified spectrophotometry UV-Vis analysis by composing a calibration curve of a series of LF concentrations. 

#### 2.2.11. Antibiotic Potency Study

The antibiotic study was performed by dissolving the samples in several pH buffer solutions, 1.2, 6.8, and 7.4, including the MIC measurement and equivalence-potency test.

- Buffer solution preparation

1. Buffer solution pH 1.2

Here, 8.63 g HCl 37% was mixed in 100 mL of distilled water. Then, the solution was diluted in distilled water with the adjustment to 1 L. Lastly, a few drops of natrium hydroxide 0.05 N were used to adjust the pH until it reached 1.2. The pH was measured using a pH meter (Mettler Toledo, Darmstadt, Germany) [15]. 

2. Buffer solution pH 6.8 

Here, 6.8 g of KH_2_PO_4_ was weighed and dissolved in 250 mL of distilled water. Then, 2 g of natrium hydroxide was dissolved in 250 mL of distilled water. After that, 250 mL of KH_2_PO_4_ solution and 125 mL of natrium hydroxide solution were mixed. The solution was diluted in distilled water with the adjustment to 1 L. Lastly, the pH was adjusted by a few drops of natrium hydroxide solution until 6.8. The pH was measured using a pH meter (Mettler Toledo, Darmstadt, Germany) [15]. 

3. Buffer solution pH 7.4 

Here, 4.303 g of Na_2_HPO_4_, 1.179 g of KH_2_PO_4_, and 9 g of natrium chloride were mixed and dissolved in 500 mL of distilled water. Then, the solution was transferred into a 1000 mL volumetric flask and diluted in distilled water with the adjustment to 1000 mL. A few drops of natrium hydroxide 0.1 M were added to adjust the pH until 7.4. The pH was measured using a pH meter (Mettler Toledo, Darmstadt, Germany) [15].

- MIC determination

MIC determination used the liquid microdilution method with Mueller Hinton broth (CLSI 2006) [16]. In this method, a microdilution tray with twofold sample-dilution steps was used. The tray was arranged by 12 columns and 8 rows of small tubes. As the tube capacity was ~300 µL and according to CLSI guidelines, the medium and bacterial inoculum were used in a small amount (~100 µL and 10 µL, respectively). Each tube was placed with 100 µL of the sample diluted under the buffer solutions (all samples LF, CA, PM, and LC each was dissolved in buffer solution pH 1.2, 6.8, and 7.4) in a series of concentrations with twofold serial dilution (at a range of 20–0.039 µg/mL of LF; PM, LC, and CA at a range of 500–0.5 µg/mL), 100 µL of medium, and 10 µL of bacterial inoculum with a concentration equivalent to the 0.5 McFarland standard (1.5 × 10^8^ colonies/mL). After that, one tube containing the 100 µL of medium-buffer solution (1:1) and 10 µL of bacterial inoculum was used as the positive control, and one tube filled with 100 µL of medium-buffer solution (1:1) without bacteria was used as the negative control. The tubes were incubated at 37 °C for 24 h. The presence or absence of bacterial growth was compared to the positive control. The minimum inhibition concentration (MIC) was obtained by the tube visual observation using a magnifying glass (Insten Magnifying Glass 10× Handled, Hereford, UK) that did not show bacterial growth at the lowest concentration. The antimicrobial potency test was performed on *Staphylococcus aureus/S. aureus* (Gram-positive) and *Escherichia coli/E. coli* (Gram-negative). The final pH of each samples tube was measured using a pH meter. 

A fertility test was conducted to check the bacteria growth in the medium. First, the 100 µL medium was mixed with buffer solution pH 1.2, 6.8, and 7.4 in a ratio (1:1) into a test tube. Next, one tube of 100 µL of culture medium—buffer (1:1) with 10 µL of bacterial inoculum (positive control), and one tube of 100 µL of culture medium—buffer (1:1) without bacterial inoculum (negative control), were prepared. Then, 10 µL of bacterial inoculum was inoculated to each medium with the corresponding buffer and incubated for three days at 37 °C. Finally, the turbidity of the tube was checked visually using a magnifying glass to observe the bacterial growth.

- Equivalence-potency test

The agar diffusion method with Mueller Hinton medium was used in the potency test. First, Mueller Hinton medium was poured into a Petri dish and inoculated with 100 µL of bacterial inoculum of equal turbidity with a 0.5 McFarland standard. Then, 3–6 metal cylinders with a diameter of 6.0 mm were placed on the agar plate. Next, five concentrations of LF standard solution (LF dissolved in the buffer solutions) were prepared to compose a calibration curve by plotting the inhibition diameter toward the log concentration (log C) of the LF solution in the three kinds of pH media. The calibration curve was used to determine each sample’s equivalence-potency (µg/mL) by substituting the inhibition zone diameter into the Y variable in the regression equation to check the linearities. Next, as much as 100 µL of each solution with appropriate concentrations of 10.24, 12.8, 16, 20, and 25 µg/mL (named S1–S5) were filled into the metal cylinders. The Petri dishes were then incubated at 37 °C for 24 h. After the incubation period, the inhibition zone diameters were measured. Finally, the potency of LC and PM equal to the middle concentration (S3), 16 µg/mL LF in all buffer solutions, was determined with the same method and compared [17].

#### 2.2.12. Statistics

All experiments were performed in independent triplicate trials. The results were presented as the mean of measurement data. Curves were prepared using Microsoft Excel (Microsoft Corp., Redmond, WA, USA).

## 3. Results

### 3.1. Molar Ratio Determination

**Figure 2:**
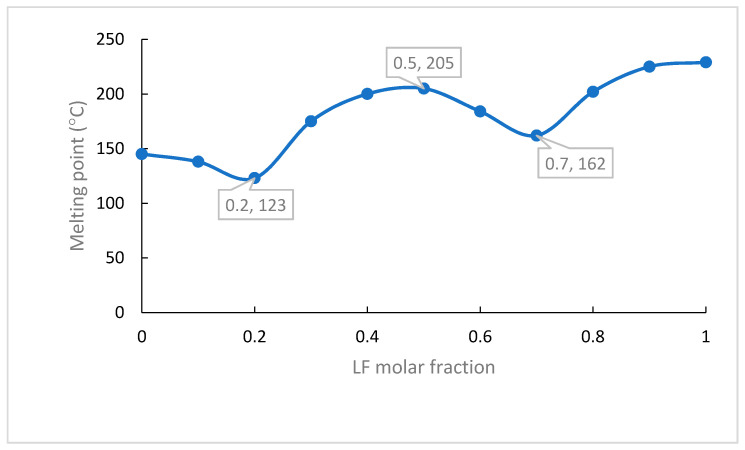
Phase diagram of LF-CA binary system.

A binary phase diagram was the first tool to determine the stoichiometric molar ratio of LF and CA in their multicomponent system [18]. The melting points versus the LF molar fraction were plotted in a curve, as shown in Figure 2, which exhibited a “W” pattern, indicating a multicomponent formation [19,20]. Based on the diagram phase profile, the new phase may be composed from the molar fraction of LF 0.2 to 0.7. Hereafter, the LC system was prepared in the stoichiometric balance of 1:2, 1:1, and 2:1, using fast and slow evaporation methods. As a result, we found a new stable phase of LC from the LF-CA mixture (1:1).

### 3.2. LC Preparation and Characterization

**Figure 3:**
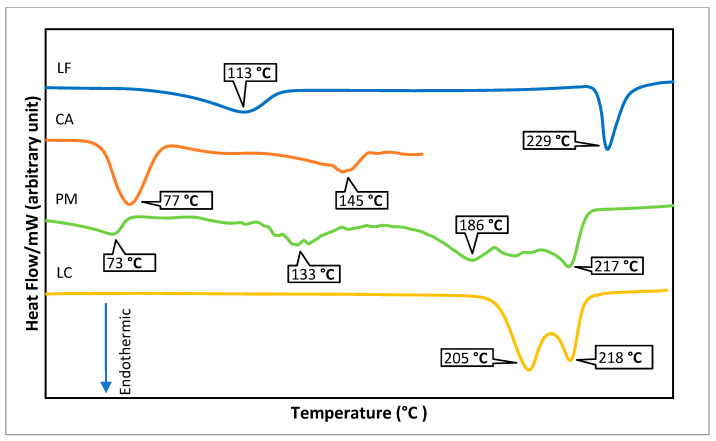
DSC thermogram of LC (1:1) compared to the starting materials, levofloxacin (hemihydrate), citric acid (dihydrate), and their physical mixture (PM).

LC obtained from the fast and slow evaporation produced crystal/white powders, resulting in similar electrothermal, DSC, and PXRD data, named LC. The electrothermal measurement of LC showed a melting point at 205 °C, followed by an oxidation point of 218 °C (observed to be burnt and become dark chocolate/black irreversibly). These data were confirmed with the DSC thermogram in Figure 3. The parent antibiotic, LF, released the water molecule, as it was a hemihydrate, at 113 °C, by melting 229 °C. CA (in its dihydrate form) was unhydrated at 77 °C, and melted with decomposition after 145 °C. PM was indicated to contain hydrates, which was shown by an endothermic peak at 73 °C. The evidence of two melting points at 133 and 186 °C represents the starting materials, and after that, PM decomposed after 217 °C. Meanwhile, both electrothermal data and the DSC thermogram indicated that the LC melted at 205 °C and decomposed after 218 °C, totally different from the starting materials and PM.

**Figure 4:**
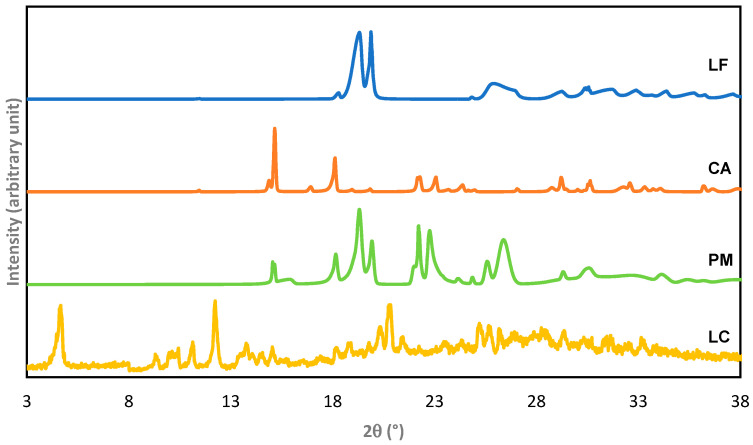
Powder X-ray diffractogram of levofloxacin citrate (LC) compared to the starting materials, levofloxacin (LF)—hemihydrate, and citric acid (CA)—dihydrate, and a physical mixture (PM) of LF and CA.

Next, the diffractogram of LC compared to LF and CA is presented in Figure 4. The diffractogram of LC depicts the new distinctive peaks at 2θ = 4.7°, 9.42°, 11.24°, 12.52°, and 20.88°, which is different from LF (in hemihydrate form) and CA (in dihydrate form). Meanwhile, PM’s diffractogram depicts a combination pattern of LF and CA. The new diffraction pattern of LC confirmed that a new solid phase was formed, which should then be structurally determined by FTIR, NMR, and SCXRD. 

### 3.3. Structural Study

**Figure 5:**
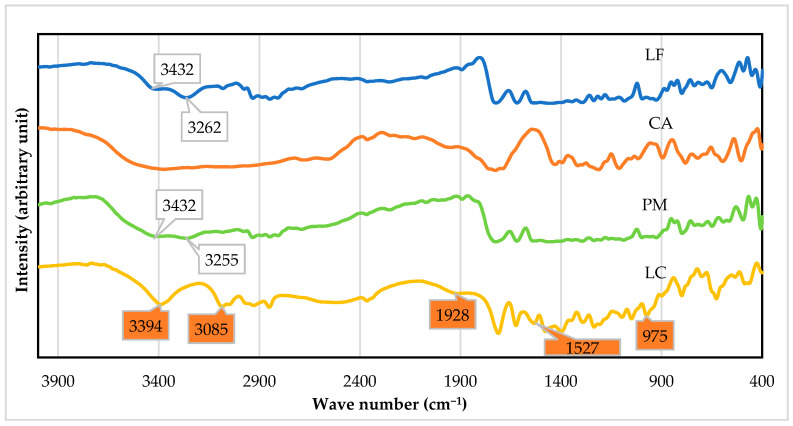
FTIR spectra of levofloxacin-citrate (LC) compared to levofloxacin (LF), citric acid (CA), and physical mixture (PM) of levofloxacin and citric acid. The new bands are depicted in the orange marks.

The structural study was performed using FTIR, ^1^H NMR, and SCXRD. FTIR was performed to analyze the intermolecular interaction in the new structure. Figure 5 depicts the compilation of LC’s spectra compared to the starting materials and their physical mixture (PM). First, the -OH broad bands of CA dihydrate were reduced. Next, the spectra of LF at 3432 and 3262 cm^−1^ were replaced by 3394 and 3085 cm^−1^ in LC, respectively, reflecting the change in N3 of the methyl piperazine group and carboxylic stretching, as the 1928 and 1527 cm^−1^ bands, respectively. In addition, the appearance of the 1000–900 cm^−1^ band reflected the -OH bending in the new interaction. The spectra changes revealed a new solid phase with less water, which involved amine with carboxylic functional groups. Meanwhile, in PM, the -OH bands were still shown as broadband, and the signals were similar to the starting materials. Based on these spectra, the interaction between the starting was significantly detected.

**Figure 6:**
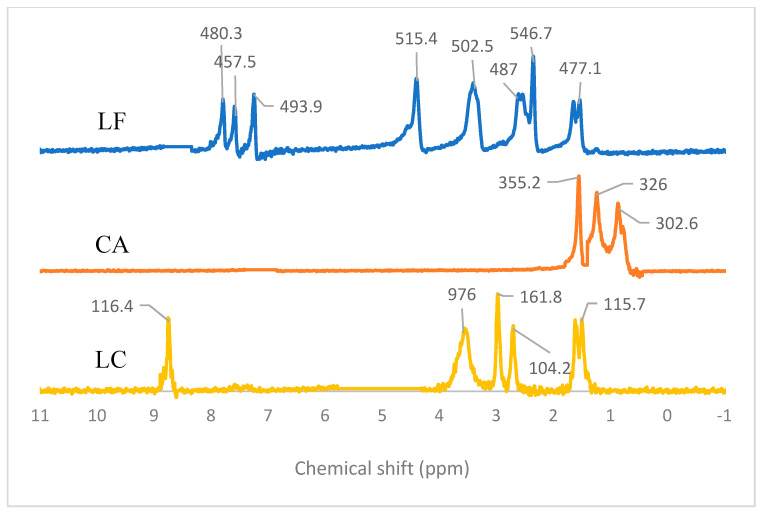
^1^H NMR spectra of levofloxacin (LF) and citric acid (CA) in CDCl_3_ solution, and levofloxacin-citrate (LC) in D_2_O.

**Figure 7:**
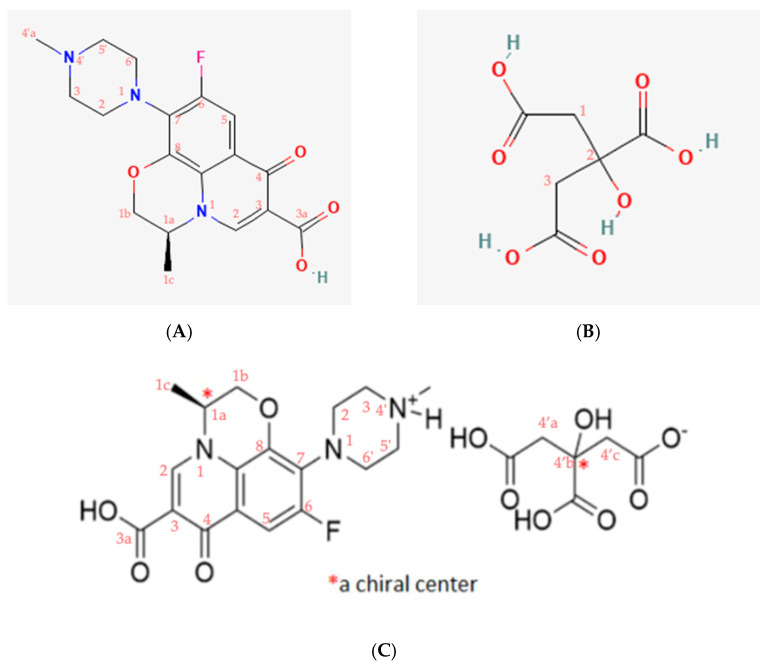
Numbering molecule structure of (**A**) levofloxacin/LF, (**B**) citric acid/CA, and (**C**) levofloxacin citrate/LC.

The structure elucidation was continued by NMR analysis. ^1^H NMR was used for 2D structural determination by characterizing the hydrogen-1 nuclei within the molecules of LC. Figure 6 depicts the ^1^H NMR spectra for LF, CA, and LC. The numbering molecule structure can be seen in Figure 7. The LF spectra showed eight signal peaks, which indicate eight types of protons. At 7.9, 7.6, and 7.3 ppm, a singlet signal appeared, which refers to the 5-H proton or aromatic group due to the coupling with the F atom in position 6. Next, a sharp singlet appeared at 4.5 ppm, referring to the 2-H proton in positions 2, 3, 5′, and 6′. Meanwhile, the duplet signal at 3.5 ppm represents the 1b 2-H protons. The signals at 2.7 and 2.4 can be attributed to the 4′a and 1c methyl groups, respectively. Lastly, at 1.8 ppm, the duplet signals that appeared can be attributed to the 3a carboxyl groups. 

On the other hand, CA spectra showed three types of protons, deemed from the three signals that appeared. At 1.7, 1.3, and 0.8 ppm, the singlet signal represents 1, 2, and 3 carboxyl groups; the 1 and 3 of 2-H protons; and the 1-H proton of position 2, respectively. The LC spectra indicate five types of protons. The singlet signal at 8.75 ppm refers to the aromatic group due to the coupling with the F atom in position 6. Next, the singlet signals at 3.6, 3, and 2.8 ppm can be attributed to 1-H of the 4′amino group, 4′b hydroxyl group, and 5-alkene, respectively. Next, the carboxyl groups of the 3a, 4′a, 4′b, and 4′c positions were observed at 1.5 ppm. The different solvents used due to the change in solubility property of LC from its starting materials supported the expectation that this new salt was combined successfully from antibiotic-antioxidant and became more polar than LF. Hereafter, the interpreted spectra of LC following the FTIR data were continued by 3D structure determination using SCXRD.

**Figure 8:**
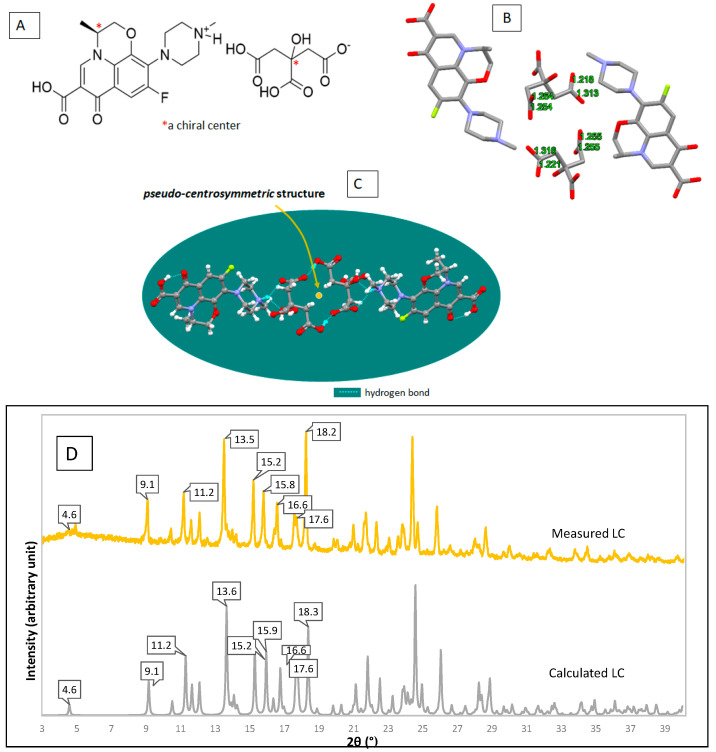
Empirical structure of levofloxacin-citrate/LC (**A**); the different C-O distance of ionized citric acid in the LC structure (**B**); ORTEP-3D structural drawing of LC with the hydrogen bonds in blue (**C**); and the calculated compared to the measured diffractogram of LC (**D**).

Next, SE using methanol 70% in water under ambient conditions (in Tokyo, 30–40% RH; 22–25 °C) produced the plate-shaped-colorless single-crystals, *which SCXRD successfully determined its crystal structure*. The structure investigation obtained data as shown in Figure 8. First, Figure 8A reveals the new salt structure scheme. Afterward, Figure 8B shows the 3D structure that depicts the different distances of C-O in CA’s carboxylic moiety, which are (1.221 Å, 1.316 Å) for COOH and (1.255 Å, 1.255 Å) for COO^–^. These data show that one of the three carboxylic groups was ionized to be a mono-anion and interacted with the positively charged methylamine of LF, explaining that LC is a salt. The site of this ionic interaction is comparable with LF-metacetamol, but with the higher energy, because the difference of pKa in that previous multicomponent structure is not enough to support ionization, only producing the hydrogen bonding [4]. Thus, the LF–CA interaction could be expected to compose a more stable compound toward lighting. 

Furthermore, Figure 8C points to the pseudo-centrosymmetric LC structure, with the CA layer replacing the LFH’s water molecule. Lastly, Figure 8D confirms the similarity between the measured and calculated diffractogram, which depicts the specific peaks at 2θ = 4.6, 9.1, 11.2, 13.5, 15.2, 15,8, 16.6, 17.6, 18.2°. 

Afterward, Table 1 shows the crystal data of LC, revealing that it has a triclinic crystal system with a volume of 1236.0 Å^3^. As a comparison, LFH was reported to have a monoclinic system with a volume of 3355.7 Å^3^ [21]. 

### 3.4. Stability Data toward Humidity and Lighting 

The color change from yellowish white of LF to the bright white powder of LC identified the crystal structure change. Afterward, from the organoleptic observation, this organic salt antibiotic seemed stable toward sunlight and humidity due to no color change during the investigation for one month in open air under ambient conditions (25 ± 2 °C/70–75% RH); meanwhile, LF’s color became darker/intense yellow. 

**Figure 9:**
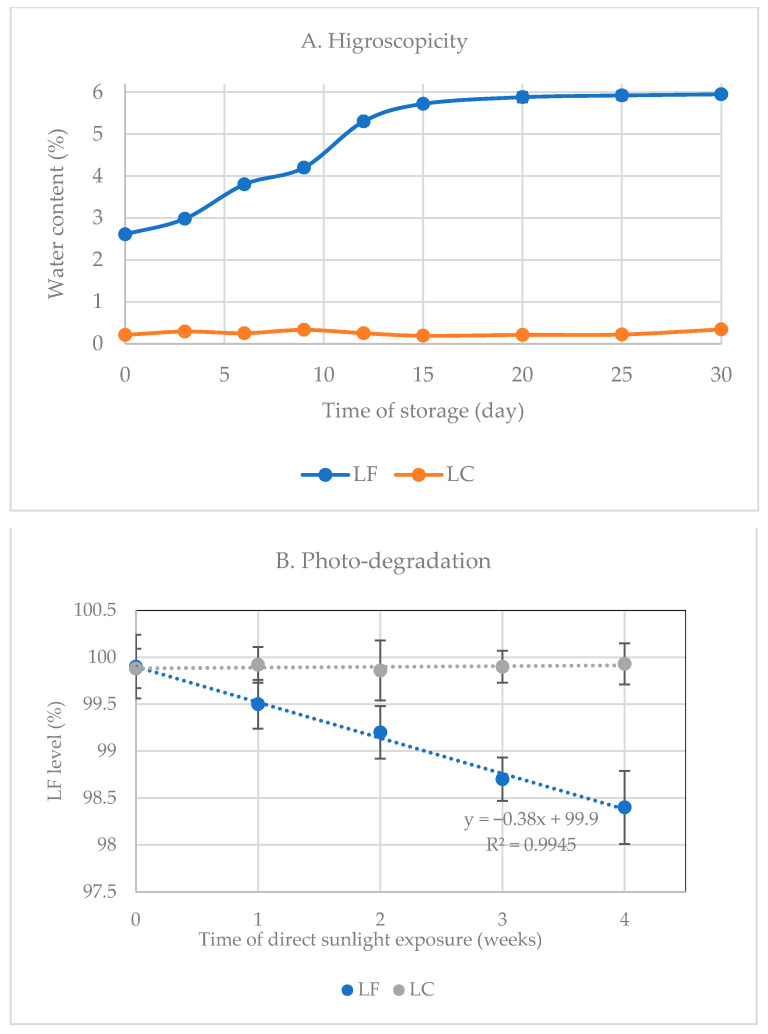
The curves of hygroscopicity test data (**A**) and photo-degradation curve (**B**) of levofloxacin (LF) and levofloxacin citrate (LC).

Based on the crystal structure in Figure 8, the new organic salt phase formed the layer of LF and CA in a pseudo-symmetric system. Even though the hydrophilicity of LF elevated in this new interaction, the hygroscopicity of LF inversely decreased. As a result, CA was seen in the inner layer, replacing the LF water and forming a denser and more compact lattice structure. Hereafter, it did not provide the space for water adsorption, altering the surface behavior changes. PXRD measurement indicated that LF hemihydrate transformed to the monohydrate form, which equaled 5.5%. This means that 0.5% moisture was still entrapped in the crystal, as shown in the thermogram in Figure 9A. 

Next, as stated in the Introduction, LF is sensitive to lighting, which accelerates its oxidative degradation. However, studies mainly reported LF instability in the solution. One of the degraded products is levofloxacin-*N*-oxide [22]. Previously, the electrothermal analyzer could visually observe the oxidative degradation, which recorded that LF turned brown and dark irreversibly after melting. The photo-stability test for one month revealed significant differences between LF and LC, as presented in Figure 9B. There was a content decrease of ~1.5% (from 99.9–98.4%) during that period, with a darker yellow appearance of LF. Until one month of investigation, the photo-degradation kinetics of LF exhibited zero-order kinetics. Meanwhile, LC’s appearance was still in bright-white color, in line with its constant level. 

**Figure 10:**
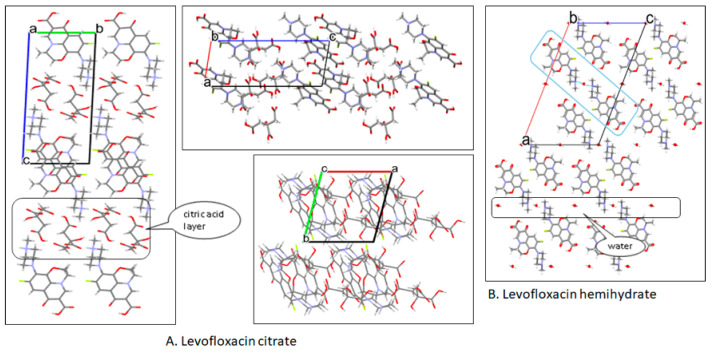
Crystal packing of levofloxacin citrate from a, b, and c view-sites compared to LF-hemihydrate as the parent compound.

Based on the crystal structure in Figure 10, CA covered the packing system, where the CA was positioned outward of LF within the crystal arrangement. This structure caused the CA to protect LF against oxidation because CA is a well-known antioxidant agent. Furthermore, the bonding of CA’s carboxylic with the N-methyl piperazine group of LF, which is strongly expected to involve oxidative degradation, preserves this compound from decomposition [4]. 

This phenomenon is similar to the reported hygroscopicity and photo-stability reduction of LF after a reaction with metacetamol. The stability improvement is comparable to the reported LF—metacetamol, which also involved N3 of the *N*-methyl piperazine in the molecular interaction [4]. However, LC can be predicted to be more stable than that of the previous multicomponent because the energy of an ionic interaction is higher than that of the neutral one. In addition, compared with metacetamol [4], CA has a higher acidity, antioxidant activity, and antimicrobial effect, all contributing to the stability and potency, which is challenging to investigate further.

### 3.5. Antibiotic Potency Test 

The microbial test was performed to ensure that the salt formation did not decrease LF’s antibiotic potency. Some reports explained that LF’s strength depends on the pH value of its environment. Hence, the antimicrobial activity was tested by dissolving each sample in several buffer solutions: 1.2 (gastric condition model), 6.8 (intestinal condition), and 7.4 (skin/plasma condition) [23,24]. In addition, the fertility test method was performed to examine the influence of pH on bacteria growth. Based on the test result displayed in Table 2, at pH 6.8 and 7.4, bacterial growth was observed well for *S. aureus* and *E. coli*, without any bacterial growth inhibition.

Meanwhile, in the medium pH 1.2, no bacterial growth was detected, showing that both bacteria could not grow under that condition. This phenomenon was caused by the neutrophil character for *E. coli* and *S. aureus*, produced under low pH conditions. Therefore, it has been reported that the optimum pH for growing those bacteria should be close to 7 (neutral), and the minimum is 4.6 [25].

For MIC determination, the concentration range of CA was built differently from the other samples because CA did not exhibit any inhibition growth in the range of 20–0.039 µg/mL, suggesting that CA needs a higher concentration for it to show antimicrobial growth activity. Hence, the concentration range of CA was prepared higher (in a range of 500–0.5 µg/mL) than the other samples. The MIC test resulted in data in Table 3, complete with the final pH of the mixture in the tube tests. The final pH of the LF sample’s solution is similar to the buffer pH due to the amphoteric structure of LF. On the other hand, CA significantly changed the medium buffer’s pH, from pH 6.8 and 7.4 to pH of 5.24 and 6.30, respectively. The solution produced a similar final pH due to LC, and PM similarly dissociated into LF and CA. The pH of LC and PM solutions became lower than LF’s due to the existence of CA, which was 6.65 (from pH 6.8) and 7.25 (from pH 7.4), respectively. 

Furthermore, Table 3 shows that PM and LC produced almost equal MIC values, ~2-fold of LF alone. This result is predicted due to the microbial activity contribution of CA, which has been reported to exhibit a weak antimicrobial activity against bacteria such as *E. coli* and *S. aureus* [26]. 

The antimicrobial activity of CA has been reported to be pH-dependent. CA was reported to be more active under an acidic environment [27], i.e., pH 3.1–4.7, which supported CA to stay as an undissociated compound [28,29]. In this form, CA freely passes through the bacterial membrane, decreasing the internal cell pH, causing the dissociation of membrane transport and structural damage. Another reference stated that CA showed the maximum antimicrobial activity at pH 9.5 because it dissociated completely into tribasic form. That tribasic species is harmful to the bacteria’s membrane. 

As published, CA is less effective in the neutral pH because it can dissociate into dibasic form, which may protect bacteria by acting as carbon sources and chelating the ionic metal in the membrane, replacing the harmful effect of the tribasic form [30,31]. In more detail, CA was found as a monobasic ion at pH < 4, became a dibasic form at pH 4–6.4, and then fully deionized to the tribasic state at pH > 7.5 [32]. In line with the references, Table 3 depicts that the final pH of CA alone after dissolving in buffer pH 6.8 and 7.4 were 5.24 and 6.30, respectively. Therefore, CA can be predicted in the dibasic form under those conditions and acts as a protective agent to the bacteria’s membrane. Hence, it showed a minor antibacterial activity, with the MIC > 500 µg/mL against *S. aureus* and 250 µg/mL against *E. coli*. Hereafter, the lower MIC or the increased potency of LC and PM can be predicted as a synergism effect of the LF-CA mixture, with the final pH 6.65 and 7.25, higher than those of CA solutions, which may support the tribasic species formation.

**Figure 11:**
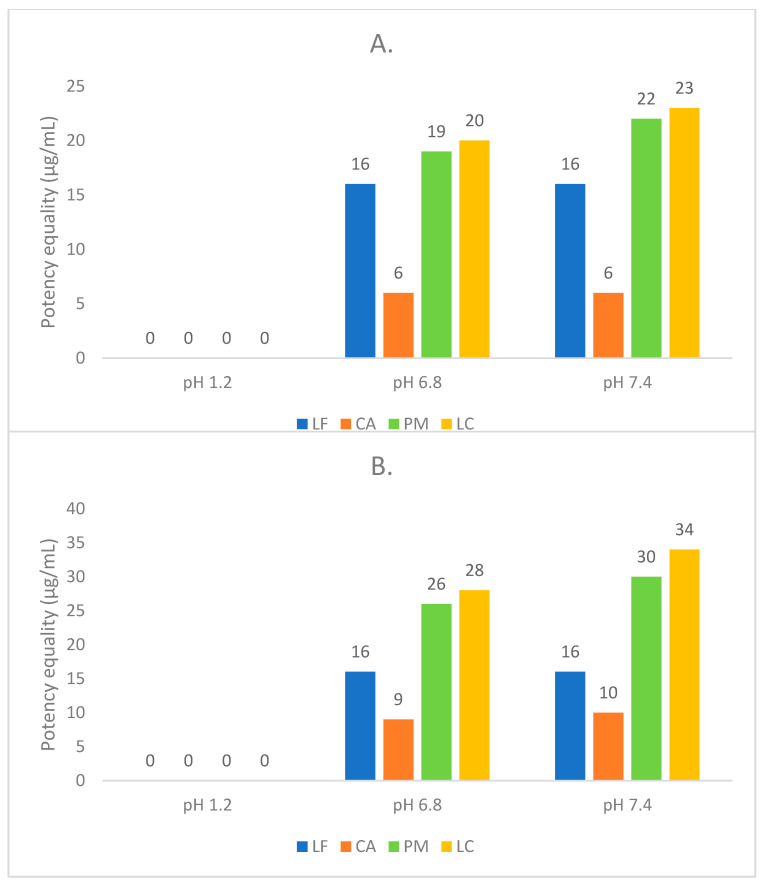
Antibiotic potency comparison against *Staphylococcus aureus* (**A**) and *Escherichia coli* (**B**). Note: levofloxacin (LF), citric acid (CA), levofloxacin-citrate (LC), and physical mixture (PM) of levofloxacin and citric acid.

Next, Figure 11 depicts the potency diagram of LF, CA, PM, and LC, based on the concentration equality of the middle concentration (S3) of LF, 16 µg/mL. Next, in line with the MIC data, the equivalence-potency test also showed that the LC and PM strength was 1.5–1.75-fold that of LF under all pH conditions. For example, equal dose strengths of LC and PM against *E. coli* in the pH 6.8 were similar with 28 µg/mL and 26 µg/mL of LF, respectively, or increased 1.75-fold. 

Furthermore, both MIC and potency data showed that CA was more effective against *E. coli* than *S. aureus*. This result was caused by the Gram-negative bacteria’s lipopolysaccharide layer (LPS) producing the negative charges. Divalent cations can decrease the electrostatic repulsion between the negatively charged groups of the membrane, and the tribasic form of CA can chelate that divalent cation away. The action of CA against the divalent cation increases electrostatic repulsion, which leads to membrane destabilization and the release of LPS, finally causing structural damage [30,33]. 

Briefly, based on the experimental data obtained, LC and PM’s potency was superior to LF. Therefore, the increase in activity of LC and PM indicates a synergism activity between LF and CA, which may also be correlated with the pH increase of CA, which may elevate the tribasic portion. However, the fixed mechanism of the potency enhancement of this combination needs to be further investigated thoroughly. 

This finding is interesting to develop further in the antibiotic formulation. However, as LC is dissociated into free LF and CA in the solution phase, it is essential to determine the potential gastro-toxicity of LF and CA. LF has low toxicity because it has a long half-life in adults. The elimination half-life of LF ranges from six to eight hours in a healthy person. On the other hand, it is necessary to maintain patient hydration [34]. A patient with genetic disorders with collagen deficiency might risk gastrointestinal toxicity when taking levofloxacin orally. The gastrointestinal tract, especially layers of the stomach and intestine walls, is arranged by collagen. As levofloxacin might induce collagen degradation, it can cause collagen deficiency and gastrointestinal perforation [35]. The dose of levofloxacin that might lead to gastrointestinal adverse effects is about 250–500 mg [36]. 

On the other hand, citric acid can delay gastric emptying in fasting patients. A high concentration of CA in the gastric acid can decrease the pH of the duodenal until pH value 6, which stimulates the bicarbonate and secretin release that can cause the delayed neutralization of the gastric acid [37]. As CA can delay gastric emptying, it increases the half-life of the levofloxacin in the stomach, which can increase the risk of gastro-toxicity. Afterward, CA can interfere with preserving the microbial balance in the gastrointestinal tract. This occurrence is because CA can lower the pH of the intestines. As a result, the number of pathogenic bacteria in low pH will increase, and nonpathogenic bacteria will reduce [38]. 

Last but not least, this research showed that LC produced from LF and CA modulated the stability and antibiotic potency of LF. Hereafter, this antibiotic-antioxidant multicomponent system is appropriate for dosage formulation, such as tablets, capsules, and topical preparation.

## 4. Conclusions

In this experiment, LF was combined successfully with an antioxidant, CA, named levofloxacin-citrate (LC). The new solid system melted at ~205 °C followed by degradation at 218 °C and had distinctive diffraction peaks at 2θ = 4.7°, 9.42°, 11.24°, 12.52°, and 20.88°. Furthermore, FTIR observed new interaction bands, and a different pattern of ^1^H NMR spectra confirmed LC formation. Finally, the new finding phase was structurally determined by SCXRD three-dimensionally as a salt composed by the interaction between the carboxylic moiety of CA and *N*-methylamine of LF (1:1). Interestingly, LC improved the hygroscopicity and photostability properties, in addition to increasing the antibiotic potency of LF ~1.5–2-fold. Hereafter, this novel antibiotic-antioxidant salt is a promising pharmaceutical candidate for dosage formulation.

## Figures and Tables

**Table 1:** Crystal data of LC.

Structure Name	Levofloxacin Citrate
Empirical formula	C_24_ H_28_ F N_3_ O_11_
Formula weight	553.49
Crystal system	Triclinic
Space group	*P*1
*a*/Å	7.61565 (17)
*b*/Å	8.63748 (14)
*c*/Å	19.5657 (4)
*α*/°	90.2939 (15)
*β*/°	99.2176 (18)
*γ*/°	103.1494 (16)
*V*/Å^3^	1235.96 (5)
*Z*	2
Density (calculated)	1.487 g/cm^3^
*R*/%	3.12
CCDC Deposition Number	2161971

**Table 2:** Fertility testing data of *S. aureus* and *E. coli* in pH 1.2, 6.8, and 7.4.

Observation Days	Fertility Test					
	*S. aureus*			*E. coli*		
	pH 1.2	pH 6.8	pH 7.4	pH 1.2	pH 6.8	pH 7.4
1	−	+	+	−	+	+
2	−	+	+	−	+	+
3	−	+	+	−	+	+

Note: (+) bacterial growth, (−) no bacterial growth.

**Table 3:** Minimum inhibition concentration (MIC) of levofloxacin citrate (LC) compared to levofloxacin (LF) and physical mixture (PM) of levofloxacin and citric acid.

Sample	MIC toward Bacteria under Different pH											
		*S. aureus* (9.7 × 10^8^ Colony/mL)						*E. coli* (1.3 × 10^8^ Colony/mL)				
	pH 1.2		pH 6.8		pH 7.4		pH 1.2		pH 6.8		pH 7.4	
	MIC (µg/mL)	Final pH	MIC (µg/mL)	Final pH	MIC (µg/mL)	Final pH	MIC (µg/mL)	Final pH	MIC (µg/mL)	Final pH	MIC (µg/mL)	Final pH
LF	0	1.2	0.1560	6.78 ± 0.02	0.1560	7.36 ± 0.08	0	1.2	0.1560	6.78 ± 0.02	0.1560	7.36 ± 0.08
CA	0	1.19 ± 0.002	>500	5.24 ± 0.005	>500	6.30 ± 0.02	0	1.19 ± 0.002	250	5.24 ± 0.005	250	6.30 ± 0.002
PM	0	1.2	0.0780	6.65 ±0.01	0.0780	7.25 ± 0.007	0	1.2	0.0780	6.65 ± 0.01	0.0780	7.25 ± 0.007
LC	0	1.2	0.0780	6.65 ± 0.01	0.0780	7.25 ± 0.007	0	1.2	0.0780	6.65 ± 0.01	0.0780	7.25 ± 0.007

Note: levofloxacin (LF), physical mixture (PM) of levofloxacin and citric acid, levofloxacin citrate (LC), and minimum inhibition concentration (MIC). *n* = 3. The agar medium was neutral with pH ~7.

## Data Availability

CCDC 2161971 contains the supplementary crystallographic data for this paper. The data can be obtained free of charge from The Cambridge Crystallographic Data Centre via www.ccdc.cam.ac.uk/structures.

## Abbreviations

API	Active pharmaceutical ingredients
CA	Citric acid
DSC	Differential scanning calorimetry
FTIR	Fourier transform infrared
FE	Fast evaporation
LF	Levofloxacin
LFH	Levofloxacin hemihydrate
LC	Levofloxacin citrate
MIC	Minimum inhibition concentration
NMR	Neutron Magnetic Resonance
PM	Physical mixture
PXRD	Powder X-ray diffractometry
RH	Relative humidity
SE	Slow evaporation
SCXRD	Single-crystal X-ray diffractometry
